# Early absorption sources of caffeine can be a useful strategy for improving female table tennis players-specific performance

**DOI:** 10.1080/15502783.2023.2282051

**Published:** 2023-11-16

**Authors:** Sepideh Pirmohammadi, Mohammad Hemmatinafar, Javad Nemati, Babak Imanian, Mohammad Hassan Abdollahi

**Affiliations:** Department of Sport Sciences, Faculty of Education and Psychology, Shiraz University, Shiraz, Iran

**Keywords:** Table tennis, caffeinated gum, coffee mouth rinsing, explosive power, female athletes

## Abstract

**Background:**

The consumption of fast absorption sources containing caffeine, such as caffeinated gum and coffee mouth rinsing, has been considered a practical nutritional strategy among athletes. Therefore, this study aimed to determine the effect of early absorption sources containing caffeine on the performance of female table tennis players.

**Method:**

Eighteen female table tennis players randomly participated in this randomized, double-blind, and crossover designed study. Before starting the test, the participants attended a familiarization session. In each test session, participants were randomly assigned to one of three conditions including chewing caffeinated gum (CG, *n* = 6), coffee mouth rinsing (CMR, *n* = 6) and placebo capsule (PLA, *n* = 6). All participants consumed caffeine with an average dose of ∼3 to 4.5 mg·kg^−1^. The one-week interval was considered a washout period for each condition. Each test session included measurement of functional, skill and cognitive tests. Skill tests included serve, forehand drive, backhand push and counter tests. The Cognitive function measured by color recognition test, and functional tests included agility, hand movement speed, the explosive power of the upper body and lower body, hand-eye coordination and hand grip strength tests. The collected data were analyzed (with SPSS Windows software) by repeated measure ANOVA analysis and Bonferroni post hoc test at *P* ≤ 0.05 level.

**Results:**

The findings of the present study illustrated that CG and CMR increased significantly agility and reduced the amounts of errors in the cognitive test compared to PLA (*p* < 0.05), While there was no significant difference between CG and CMR (*p* > 0.05). Also, CG and CMR compared to PLA and CMR compared to CG rose significantly hand movement speed and movement speed (*p* < 0.05), and CMR compared to PLA increased significantly hand-eye coordination, isometric hand strength, service accuracy and forehand drive (*p* < 0.05). However, CG compared to PLA and CMR had no significant effect on hand-eye coordination, isometric hand strength, service accuracy and forehand drive (*p* > 0.05). In addition, CG and CMR enhanced significantly the explosive power of the lower body compared to PLA (*p* < 0.05), While there was no significant difference between CG and CMR (*p* > 0.05). Also, CG and CMR compared to PLA and CG compared to CMR had no significant effect on the explosive power of the upper body, backhand, and counter skills (*p* > 0.05). Furthermore, CG increased significantly accuracy in the service test compared to PLA (*p* < 0.05).

**Conclusion:**

According to the results, it seems that early absorption sources of caffeine (CMR and CG) are efficient strategies for improving the specific performance of female table tennis players. However, allegedly CMR and CG have a better effect on functional and cognitive tests compared to skill tests.

## 1. Introduction

Table tennis belongs to the group of racket sports and is associated with constant changes in the intensity of the game and repetition of fast activities in a short time. During the competition, table tennis players need many physical and physiological factors such as speed, strength, cardiorespiratory endurance, agility, perceptual skills, and quick decision-making [1]. Most scientists around the world agree that table tennis is an aerobic metabolic sport that requires high endurance, often interspersed with short periods of intense anaerobic metabolism [2]. So, proper nutrition is important for success in playing table tennis and can affect the players’ ability to practice, play and recover after the activity [3]. Also, dietary components such as vitamins, minerals and supplements can be included in the diet of athletes through varied diets and can affect neuro-muscular and metabolic function [3]. In this regard, one of the energizing supplements that have attracted the attention of many athletes and coaches is caffeine [4] Caffeine is a widely utilized performance-enhancing supplement used by athletes and non-athletes alike. In recent years, a number of meta-analyses have demonstrated that caffeine’s ergogenic effects on exercise performance are well-established and well-replicated, appearing consistent across a broad range of exercise modalities [5]. The mechanism of action of caffeine is the mobilization of fatty acids and the saving of muscle glycogen consumption, but new studies suggest that the main mechanism responsible for the physiological effects of caffeine is the inhibition of adenosine receptors of the central nervous system [6]. Since caffeine is evenly distributed in the intracellular fluid, it can affect the central nervous, respiratory, cardiovascular and skeletal-muscular systems [7]. Furthermore, in recent years, sources other than coffee and caffeine tablets that are usually consumed, such as caffeinated gums, mouth rinsing, aerosols, powders, energy-generating gels, etc., have received more attention [8].

As mentioned, one alternative source of caffeine is mouth rinsing with coffee. Coffee has a dark color and bitter taste and has a stimulating effect on humans due to its caffeine content [9,10]. It has also been found that the amount of caffeine in a typical shot of espresso coffee (1 ounce) typically contains 65 to 85 mg of caffeine [10]. The results of some studies show that bitter products can increase performance and also signal to our brain that the organism is ready for action [11]. In addition, the salivary absorption of caffeine [12] has led researchers to pay more attention to the effect of rinsing the mouth with fluids to improve athletic performance. Furthermore, researches have shown that sensing nutrients in the mouth can quickly affect the neural function of the brain and its motor outputs [5]. Also, rinsing the mouth with caffeine improves exercise performance due to the activation of the sensorimotor cortex of the brain (duration 5–20 seconds) increases [8]. These findings are very valuable because it is possible to enjoy the benefits of a method such as rotating in nutrient combinations without worrying about digestive discomfort caused by taking nutritional supplements [13]. The beneficial effects of caffeine on improving sports performance are rooted in the activation of the central nervous system and are not related to metabolic aspects [13]. In fact, caffeinated solutions increase motor output and brain activity without increasing plasma caffeine levels [13,14]. Moreover, the available evidence suggests that mouth rinsing with a caffeine solution, by increasing the activity of oral receptors, improves central conduction and arousal and thus improves physical performance [13,14]. Also, the information obtained from the studies shows that the benefits of consuming carbohydrates and caffeinated drinks are similar [14]. For example, research has shown that repeated mouth rinsing with low and high doses of espresso coffee is a useful strategy for improving the specific function of male futsal players, however, higher dose coffee mouth rinsing appears to have more profound effects on performance improvement than lower doses [15].

Interestingly, an alternative method of caffeine delivery via chewing gum may provide an advantage over traditional forms of caffeine administration. Caffeine via chewing gum offers a different pharmacokinetic profile over the ingestion of caffeine in capsules, which results in an earlier increase in blood plasma caffeine concentration, usually between 5 and 15 min from intake [16]. Moreover, chewing gum allows caffeine to be absorbed directly into the bloodstream through the buccal mucosa, thereby bypassing hepatic metabolism [16], and Approximately 85% of caffeine is released within 5 minutes after chewing, and its plasma concentration reaches its peak in about 15 minutes, which is faster than caffeine capsules (between 45–60 minutes) [17]. Several studies have shown that delivering caffeine in the form of chewing gum may accelerate the rate of caffeine transfer to the blood through absorption from the buccal cavity [16,18]. Also, compared to caffeine capsules, caffeinated gums may require a shorter waiting time from consumption to the start of the training session [8]. It has been observed in research that cycling performance improves when caffeinated gum containing 300 mg of caffeine is consumed five minutes before exercise [19]. Interestingly, administration of the same dose of caffeinated gum 60 and 120 minutes before exercise reversed the observed ergogenic effects [19]. Similarly, another study found that 1–3 mg/kg of body weight of caffeine from chewing gum delayed fatigue during repeated speed cycling [20]. We could not find a study of caffeine gum and mouth rinsing with espresso coffee on Rocket Sports players’ performance. However, a study found that consuming 6 mg of caffeine 30 minutes before exercise increases efficiency in specific actions in paddle tennis players [21]. A study also observed that consuming 3 mg of caffeine per kg of body mass as an energy drink increases jumping performance and activity patterns during play in elite badminton players [22].

According to the above, the effect of different sources of caffeine and different ways of consuming it such as CG and CMR, especially on skill, cognitive function and performance of athletes, especially in table tennis players, has not been studied sufficiently. In addition, the results of the researches that have been done on this field, are contradictory. Therefore, the purpose of this research was to investigate the consumption of fast-absorbing sources of caffeine, such as mouth rinsing with coffee and caffeinated chewing gum, on the performance, skill and cognitive indicators of female table tennis players.

## 2. Methodology

### 2.1. Participants

Eighteen females with almost 3 years of participation in the Table Tennis League of Iran, voluntarily participated in this study. The demographic information of the participants is listed in Table 1. Participants had no known diseases or medical issues, no history of allergy to caffeine or coffee, and were not consuming any supplements or medications. Furthermore, the participants did not smoke, or consumed alcohol or caffeinated beverages at the time of data collection. Before the implementation of the intervention, the study procedures were explained to the participants, and consent was obtained. This study was conducted in accordance with the Declaration of Helsinki and was approved by The Human Research Ethics Committee of Shiraz University (Code: IR.US.REC.1401.038). Additionally, all participants were members of the same training camp and, their training regime was the same under the supervision of trainers. In addition, all participants were right-handed. Most players played an all-rounder style (*n* = 9), while some were offensive (*n* = 6), and few were defensive (*n* = 3).

**Table 1.** The anthropometric data of participants.

Characteristic	Mean ± SD (*n* = 18)
Age (years)	20.50 ± 3.05
Height (cm)	165.16 ± 5.00
Weight (kg)	56.83 ± 6.50
BMI (kg/m^2^)	20.83 ± 2.44

## 3. Study design

**Figure 1.**
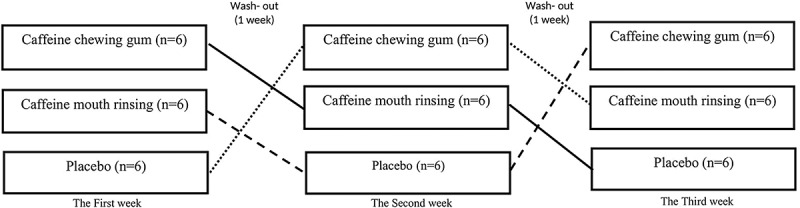
Cross-over and double-blind study design in three conditions.

This study was carried out in a randomized, cross-over, placebo-controlled, and double-blinded manner (Figure 1). Before the beginning of the investigation, the participants attended a familiarization session. During this session, participants were familiarized with all testing protocols and procedures. In each test session, the participants were randomly placed in one of the three conditions including: i) Chewing caffeinated gum (CG, *n* = 6), ii) Coffee mouth rinsing (CMR, *n* = 6), iii) Starch capsule as a placebo (PLA, *n* = 6). Forehand drive, backhand push, service and counter tests have been used for skill tests, color recognition test has been used for the cognitive test, and agility test, hand movement speed, movement speed, isometric hand strength, alternate hand wall toss test, the explosive power of the upper body and lower body muscles were measured for functional tests. Participants were provided with a list of dietary sources of caffeine and asked to refrain from consuming these 24 hours before each exercise test session. Also, a one-week interval was considered a washout period for each condition, and each test session measured functional, skill, and cognitive tests.

### 3.1. Supplementation

**Figure 2.**
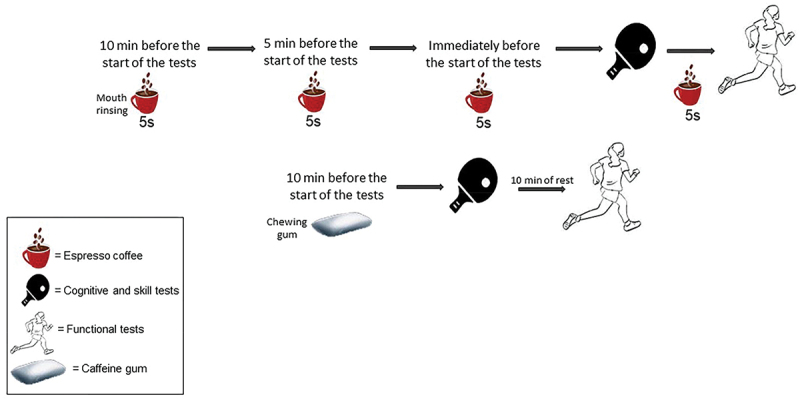
The protocol of taking supplements and performing tests.

All caffeine doses were administered dependent on the table tennis player’s pre-study body mass. If the subject’s body mass was less than 65 kg, they consumed 200 mg of caffeine (*n* = 17) and if the body mass of the participants were more than 65 kg, they consumed 300 mg of caffeine (*n* = 1) in the form of CG [23]. In the CMR condition, participants consumed approximately 240 mg caffeine in four repeated mouth rinsing and this regime ensured all table tennis players consumed a moderate dose of ∼3 to 4.5 mg·kg^−1^. In the CG condition, participants consumed chewing gum containing 100 mg of caffeine 10 minutes before the start of the tests and then participants performed skill and cognitive tests, and after 10 minutes of rest, they performed functional tests (Figure 2). In the CMR condition, participants performed four repeated times CMR (25 mL each time for 5 s, Figure 2). They did the first time CMR 10 minutes before the start of the test and the second and third times CMR was performed 5 minutes and immediately before the start of the test, respectively. The fourth time CMR was performed 5 minutes after performing the cognitive and skill tests, and then functional tests were done. In PLA condition, participants consumed one capsule containing 5 grams of starch 45 minutes before the start of the tests (Figure 2). All trials were completed at approximately the same time of day (Between 10–11 am) and all participants were fed the same breakfast containing 350–400 kcal (64% carbohydrates, 20% protein and 16% fat) 2 hours before the exercise test session [15]. Participants were instructed to maintain their normal diet throughout the testing period, to avoid food an hour before testing, and to avoid strenuous exercise 24 hours before each trial. In addition, participants had access to drink water on a self-selected basis during the trials.

### 3.2. Dosage and type of caffeine supplements

Espresso coffee was obtained from the combination of 7 grams of ground coffee (%100 Arabica Lavazza Espresso Italiano – single shot). The amount and temperature (180–190 F) of the water, as well as the pressure (9–10 bars:900–1000 Kpa), applied to prepare coffee, were the same for all. The approximate amount of caffeine in this coffee was 60 mg and the water pressure used to make coffee was 20 bar. Also, the espresso machine (Zigma20 bar RL-333N) was the same for preparing all coffees. For the caffeine gum, the MEG – Military Energy Gum made in the USA was used, and each gum contains 100 mg of caffeine and the placebo capsule contains 5 g of starch.

### 3.3. Functional tests

Agility test (Edgren’s agility test): In which five cones are placed on the ground at a distance of one meter, and the subject stands on either side of the middle cone and moves to the right with the starting signal, until the right foot, reach the outer line or pass it. Then she goes to the left until her left foot reaches or crosses the outside line and goes to the right again and continues this action for 10 seconds. For each distance of 1 meter, the subject is given one point, and if the subject cannot keep her feet forward all the time or if she does not complete the course successfully, will not be awarded a point [24].

Hand movement speed test: There is a flat board with dimensions of at least 1 × 0.5 meters and two circular surfaces with a diameter of 20 cm whose centers are 80 cm apart on both sides of the board. Between these two circles, a rectangular sheet measuring 20 × 10 cm is pasted. In this test, the person stands in front of the test board in such a way that the feet are slightly apart from each other. Then he places his non-dominant palm on the rectangular plane and places his dominant palm diagonally on the left or right side of the circle. Upon hearing the start command, with the necessary speed and accuracy, he moves his superior hand forward and backwards on the circles to perform 25 movements or 50 times touching the circles A and B, and the time to do it was recorded [25].

Alternate Hand Wall Toss Test (HWTT): This test was used to measure hand-eye coordination, where participants had to stand behind a line 1.5 meters from the wall and hold a tennis ball in their right or left hand. The subject’s knees should be slightly bent and his feet should be shoulder-width apart. The subject throws the ball against the wall with his right hand and catches it with his left hand. Similarly, if it is thrown with the left hand, it should be caught with the right hand. With this movement, the ball is thrown several times in 30 seconds and the number of successful catches is recorded [26].

Movement speed test: 20 pieces of wood are placed on the ground at a distance of 45 cm from each other so that the total test distance is 9 meters. The athlete is placed behind the starting line, the distance from which is 22.5 cm to the first stick, he must cover a distance of 9 meters with the maximum possible speed by slightly raising his knees. When the athlete’s first foot is in the area between the first and second sticks, the coach starts the timer and stops it when one of the feet touches the ground after the last stick [27].

Explosive power of lower body muscles: Sargent’s jump test was used and all evaluations in Sargent’s jump test were performed three times in a row (1 minute of passive rest between them), and the best score was recorded as the final result [28].

Explosive power of upper body muscles: A 3 kg medicine ball was used, and the participants threw the ball three times in front of their chest, and their best record was recorded in centimeters [29].

Isometric handgrip strength: The hand dynamometry test was carried out to assess the maximum isometric force in the flexors of the fingers with a (Jamar Hydraulic Hand Dynamometer, Warrenville, IL, USA) and a range between 0 and 100 kg. Measurements were performed with the participants standing with their arms fully extended to the side without touching the body. Participants were asked to hold the dynamometer with as much force as possible and alternately move the dominant hand three times for less than 3 seconds. A rest interval of at least 60 seconds was allowed between each trial and the best record was recorded as a score [30].

### 3.4. Skill tests

**Figure 3.**
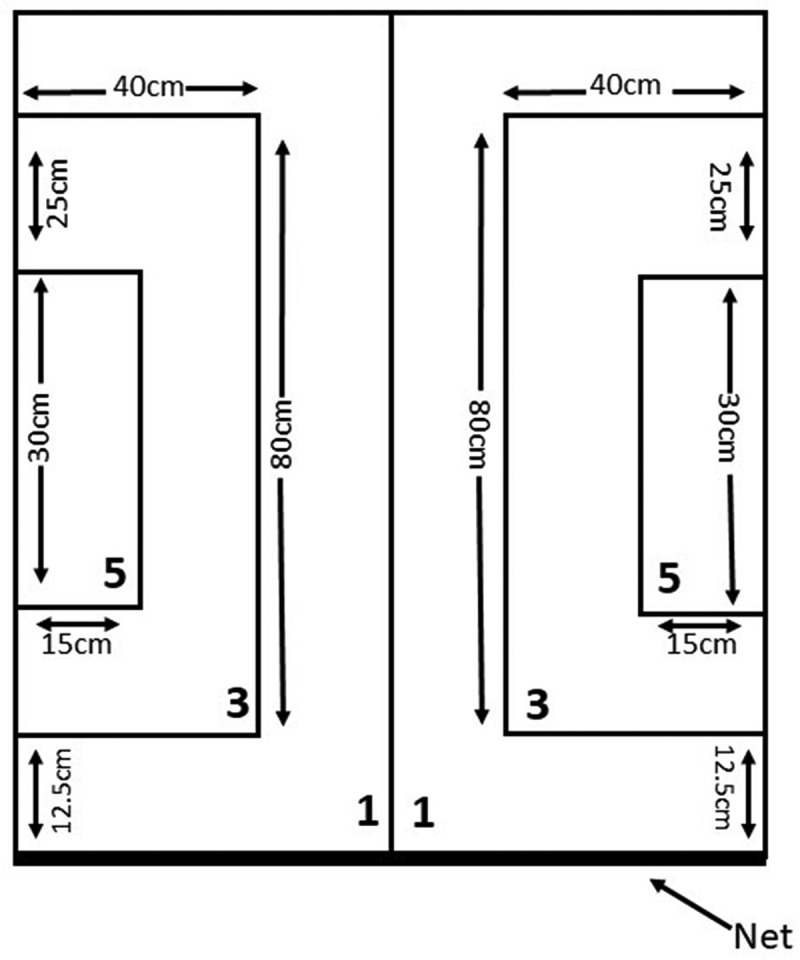
Service test on target.

Service Test: two 5-point target areas with an area of 15 × 30 centimeter (cm) on both sides of the table and at a distance of 37.5 cm from the net, and two other target areas with dimensions of 40 × 80 cm on the sideline on both sides at a distance of 12.5 cm of the net was determined (this area was worth 3 points), the remaining area of the table was worth 1 point (Figure 3) [31].

Backhand-push Test: Twine was fixed on the clamp parallel to the net assembly at a height of 20 cm above the net. The subject was instructed to warm up and practice before administering the test. The ball thrower device (Yinhe Table Tennis Robot R2 SP-17, throws the ball with the following screw: Speed: 6 m/s, Frequency: 6 Hz) toward the subject, and she has to hit backhand-push to pass the ball between the net and the twine. If the ball passes between the net and the twine, one point; if it passes over the twine, 0.5 points; and if it hits the net or goes out, no points are awarded to the subject [31].

Counter Test: The subject was asked to make the number of rallies of the alternate counter (one forehand and one backhand) at the left corner of the table with the ball thrower device (Yinhe Table Tennis Robot R2 SP-17, throws the ball with the following screw: Speed: 7 m/s, Frequency: 7 Hz) for a period of 30 seconds after sufficient warming up and practice. The ball thrower device throws 30 balls in 30 seconds. The ball thrower device throws 30 balls in 30 seconds toward the subject, and the number of balls that pass over the net is recorded as their record [31].

**Figure 4.**
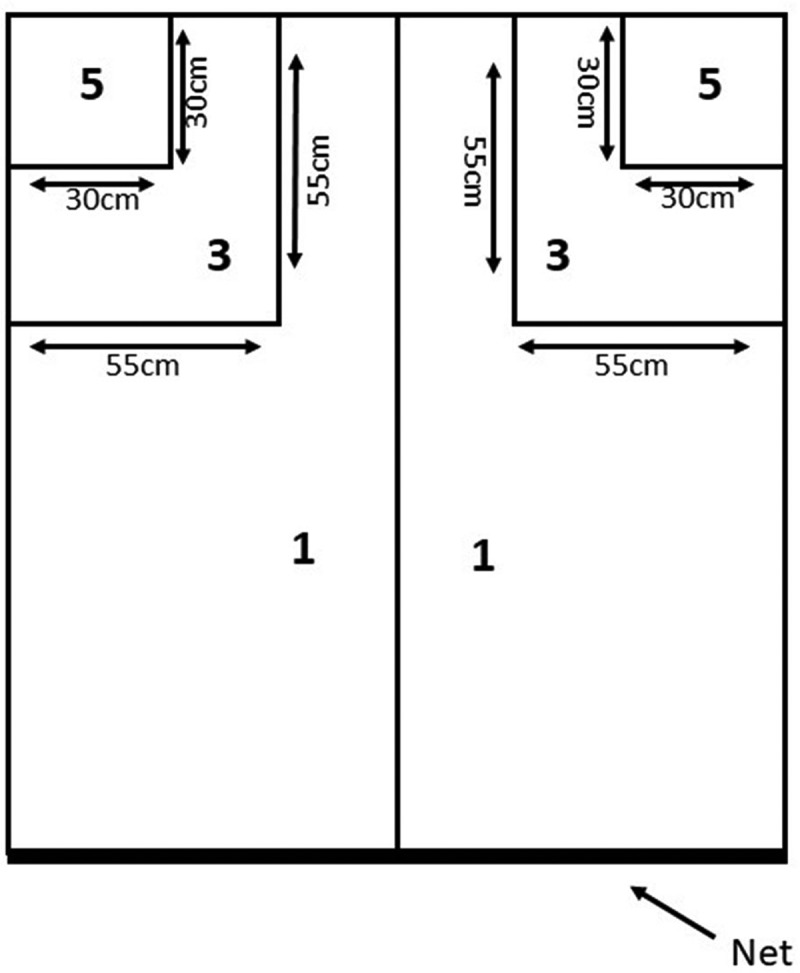
Forehand drive test on target.

Forehand Drive: Two target areas of 30 × 30 cm from the corner point of the table in both corners of a specified part of the table, and if the player’s forehand drive lands on this point, the participant gets 5 points. Two other target areas measuring 55 × 55 cm from the corner point were marked in both corners of one part of the table, which had 3 points, and the remaining parts of the table had 1 point (Figure 4) [31].

### 3.5. Cognitive test

This test examines the speed of cognitive processing in table tennis. The playground table on the opposite side of the test participant is divided according to colors and longitudinally (5 colors and equally). The participant stands on one side of the table, and the ball thrower device (Yinhe Table Tennis Robot R2 SP-17) is on the other. The device works when the start signal is given by the researcher by turning the device on, which throws the balls at a rate of 25 balls within 25 seconds (Speed: 7 m/s, Frequency: 7 Hz) and randomly in terms of direction and color, the player has to respond to those colored balls by striking it to the colored part of the table playground with the same color of the ball so that the color of the ball is perceived and hit toward the color on the table. Each color has five balls fired randomly. In order to score in this test, the player must hit the ball correctly within 25 seconds and land it in the opponent’s yard on the matching colored area. The highest possible score is 50, while the lowest score achievable is zero [32].

All Skills and cognitive tests were performed on the table (Butterfly 18) and with the racket (Timo Boll spirit, Arylate-CARBON, Japan) and ball (Double fish, ITTF V40+, China).

### 3.6. Data analysis

All data were analyzed using descriptive and inferential statistical methods. The data distribution normality was determined using the Kolmogorov-Smirnov test. Repeated measure analysis ANOVA test was used to determine the main effect on functional, skill and cognitive indicators, and Bonferroni post hoc test was used to determine pairwise differences. Data were analyzed using SPSS software (version 26, IBM-SPSS Inc., Chicago, IL, USA). The level of statistical significance was *P* ≤ 0.05.

## 4. Results

Descriptive characteristics (including mean and standard deviation) are reported in Table 2.

**Table 2.** The functional variables (mean ± standard deviation) measured in the three different experimental conditions.

Variables	PLA	CMR	CG
Agility (score)	23.22 ± 2.41	24.5 ± 1.85*	24.38 ± 2.19*
Hand speed (n)	12.19 ± 1.43	10.42 ± 1.30*	11.08 ± 1.27*^#^
Coordination (n)	26.88 ± 2.21	28.88 ± 1.99*	28 ± 1.45
Explosive power of lower body muscles (w)	1698.55 ± 49.68	1935.38 ± 491.18*	1865.11 ± 452.00*
Explosive power of upper body muscles (cm)	438.66 ± 44.76	447.72 ± 46.46	448.33 ± 61.59
Speed of movement (s)	4.16 ± .40	3.54 ± 0.23*	3.74 ± 0.22*^#^
Hand strength (kg)	53.05 ± 7.5	58.77 ± 5.01*	55.27 ± 8.30
Forehand drive (score)	15.72 ± 1.99	17.44 ± 2.5*	16.11 ± 2.58
Backhand Push (score)	28.30 ± .94	28.55 ± 0.99	28.05 ± 1.25
Serv (score)	36.33 ± 4.02	38.88 ± 3.59*	38.11 ± 4.82
Counter (n)	26.83 ± 1.33	26.94 ± 1.34	26.66 ± 1.87
Cognitive test (n)	2.11 ± 1.84	22.61 ± 1.53*	22.44 ± 1.5*

*: Significant difference compared to PLA(*P* < 0.05). #: Significant difference compared to CMR(*P* < 0.05).

**Figure 5.**
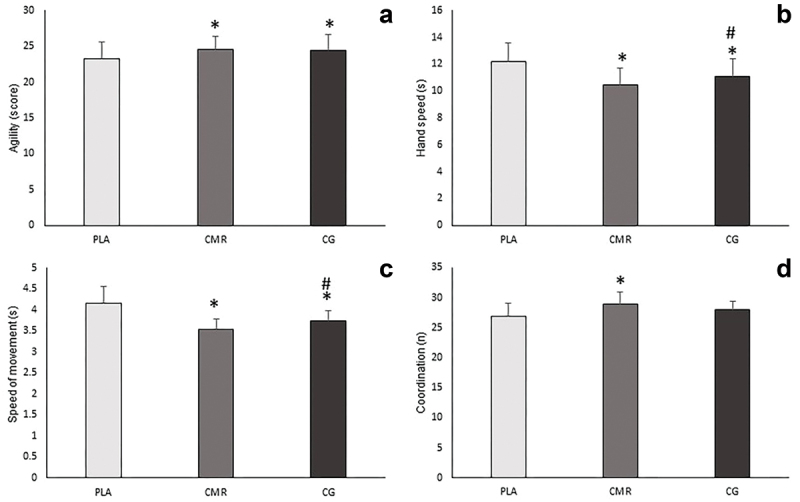
Changes in agility, hand movement, speed of movement and coordination in three conditions of this study (PLA: placebo. CMR: caffeine mouth rinsing. CG: caffeine chewing gum). (a) agility test. (b) hand speed test. (c) speed of movement test. (d) coordination test. *: significant difference compared to PLA(*P* < 0.05). #: significant difference compared to CMR(*P* < 0.05).

**Figure 6.**
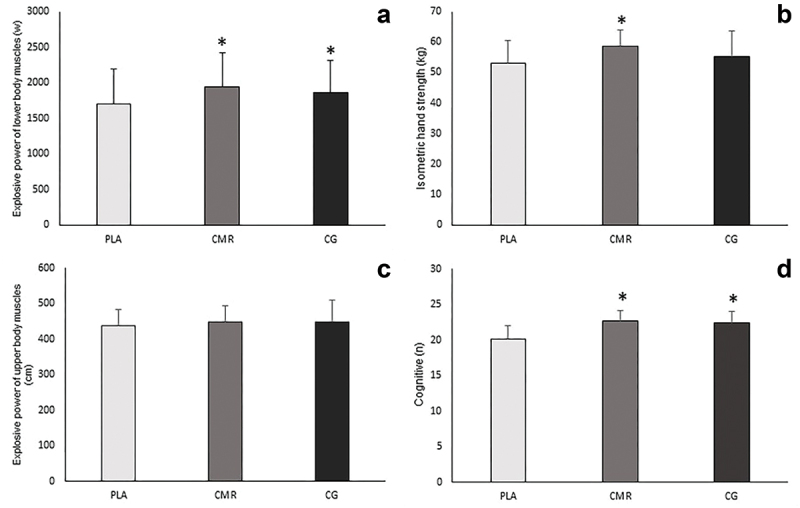
Changes in the explosive power of lower body muscles, isometric handgrip strength, explosive power of upper body muscles and cognitive test in three conditions of this study (PLA: placebo. CMR: caffeine mouth rinsing. CG: caffeine chewing gum). (a) explosive power of lower body muscles. (b) isometric hand strength. (c) explosive power of upper body muscles. (d) cognitive test. *: significant difference compared to PLA(*P* < 0.05).

**Figure 7.**
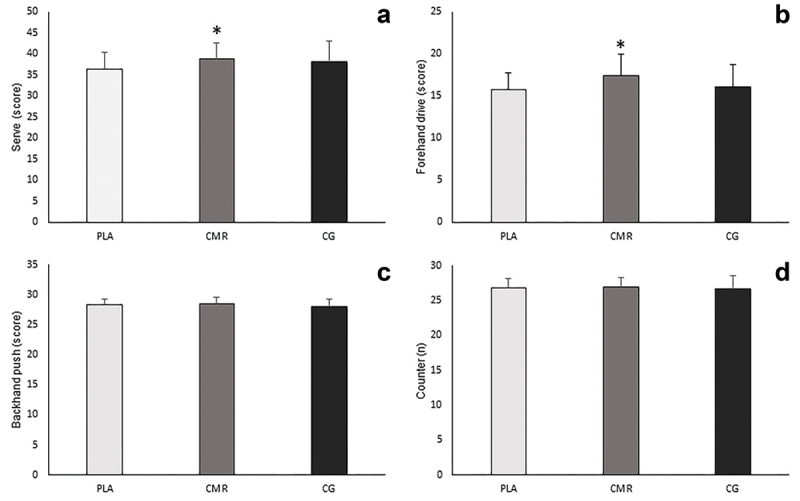
Changes in serve, forehand drive, backhand push and counter in three conditions of this study (PLA: placebo. CMR: caffeine mouth rinsing. CG: caffeine chewing gum). (a) serve test. (b) forehand drive test. (c) backhand push test. (d) counter test. PLA: placebo. CMR: caffeine mouth rinsing. CG: caffeine chewing gum. *: significant difference compared to PLA(*P* < 0.05).

The results of the analysis of variance with repeated measures showed that the main effect on agility was significant (F = 7.938, *P* = 0.005, pEta2 = 0.318). Also, the results of the Bonferroni test indicated that the agility in CG (MD = 1.167, *P* = 0.002, 95% CI [0.415–1.918]), and CMR (MD = 1.278, *P* = 0.037, 95% CI [0.067–2.488]) increased significantly compared to PLA, but there was no significant difference between CMR and CG (MD = 0.111, *P* = 1, 95% CI [-0.067–2.488]) (Figure 5(a)). Additionally, the results exhibited that there was a significant difference between the studied conditions in hand movement speed [*P* = 0.000, F = 28.232, pEta2 = 0.624], and the results of post hoc test showed that CG (MD = -1.103, *P* = 0.000, 95% CI [-1.688– -0.519]) and CMR (MD = -1.769, *P* = 0.000, 95% CI [-2.556– -0.981]) compared to PLA, and CMR compared to CG (MD = -0.666, *P* = 0.006, 95% CI [-1.149– -0.182]) significantly increased hand movement speed (Figure 5(b)). Moreover, the results showed that there was a significant difference in movement speed between the studied conditions [F = 25.016, *P* = 0.000, pEta2 = 0.595], and the movement of speed was significantly higher in CG (MD = -0.413, *P* = 0.001, 95% CI [-0.656– -0.171]) and CMR (MD = -0.614, *P* = 0.000, 95% CI [-0.889– -0.329]) than PLA, also in CMR compared to CG (MD = -0.201, *P* = 0.012, 95% CI [-0.316– -0.040]) (Figure 5(c)). Furthermore, the main effect on hand-eye coordination was significant [F = 7.893, *P* = 0.002, pEta2 = 0.317] and the accuracy in eye-hand coordination test was significantly higher in CMR compared to PLA (MD = 2.000, *P* = 0.003, 95% CI [0.642–3.358]). However, there was no significant difference between CG and CMR (MD = 0.889, *P* = 0.334, 95% CI [-0.517–2.295]), and between CG and PLA (MD = 1.111, *P* = 0.091, 95% CI [-0.138–2.361]) (Figure 5(d)). In addition, a significant difference was observed between the studied conditions in lower body explosive power [F = 17.094, *P* = 0.000, pEta2 = 0.501]. Also, the results of post hoc test showed that CG compared to PLA (MD = 166.556, *P* = 0.001, 95% CI [69.422–263.689]) and CMR compared to PLA (MD = 236.883, *P* = 0.000, 95% CI [116.781–356.886]) increased significantly the explosive power of the lower body muscles. Despite this, no significant difference was observed between CG and CMR (MD = 70.278, *P* = 0.351, 95% CI [-42.679–183.234]) (Figure 6(a)). Additionally, the results showed that there is a significant difference between the investigated conditions in the hand isometric strength [*P* = 0.001, F = 8.174, pEta2 = 0.325]. The hand isometric strength increased significantly in CMR compared to the PLA (MD = 5.722, *P* = 0.006, 95% CI [1.539–9.905]), but there was no significant difference between CG and PLA (MD = 2.222, *P* = 0.311, 95% CI [-1.209–5.654]), and between CMR and CG (MD = 3.500, *P* = 0.068, 95% CI [-0.213–7.213]) (Figure 6(b)). As well as, the main effect on cognitive function was significant [*P* = 0.005, F = 20.266, pEta2 = 0.544], and the cognitive function was significantly higher in CG (MD = 2.333, *P* = 0.000, 95% CI 1.100–3.566]) and CMR (MD = 2.500, *P* = 0.000, 95% CI [1.155–3.845]) compared to PLA, but no significant difference was observed between CMR and CG (MD = 0.167, *P* = 1, 95% CI [-0.699–1.032]) (Figure 6(d)). Additionally, the results revealed that there was a significant difference between the studied conditions in the service performance [*P* = 0.009, F = 5.502, pEta2 = 0.245], and the results of post hoc test indicated that CMR increased significantly the accuracy of service compared to the PLA (MD = 2.556, *P* = 0.005, 95% CI 0.744–4.367]), but no significant difference was observed between CG and PLA (MD = 1.778, *P* = 0.211, 95% CI [-0.655–4.221]), and between CMR and CG (MD = 0.778, *P* = 0.939, 95% CI [-1.208–2.763]) (Figure 7(a)). Moreover, the main effect on forehand drive performance was significant [*P* = 0.03, F = 6.812, pEta2 = 0.286], and the accuracy of forehand drive significantly increased in CMR compared to the PLA (MD = 1.722, *P* = 0.003, 95% CI 0.570–2.874]), but there was no significant difference between CG and PLA (MD = 0.389, *P* = 1, 95% CI [-0.919–1.697]), and between CMR and CG (MD = 1.333, *P* = 0.071, 95% CI [-0.090–2.757]) (Figure 7(b)). However, the results demonstrated that there was no significant difference in backhand push performance [*P* = 0.318, F = 1.50, pEta2 = 0.063], counter performance [*P* = 0.710, F = 0.345, pEta2 = 0.020], and the explosive power of upper body [*P* = 0.192, F = 1.735, pEta2 = 0.093] (Figures 7(c), 6(c,d)) (Table 3).

**Table 3.** Pairwise comparisons in three conditions (PLA, CMR, CG).

Variables	Condition1	Condition2	Mean Difference	Sig	95% Cl
Agility	CMR	PLA	1.278*	0.037	0.067–2.488
		CG	0.111	1.000	-0.689 – -0.911
	CG	PLA	1.167*	0.002	0.415–1.911
Hand speed	CMR	PLA	-1.769*	0.000	-2.556 – -0.981
		CG	-0.666*	0.006	-1.149 – -0.182
	CG	PLA	-1.103*	0.000	-1.688 – -0.519
Coordination	CMR	PLA	2.000*	0.003	0.642–3.358
		CG	0.889	0.334	-0.517–2.295
	CG	PLA	1.111	0.091	-0.138–2.361
Explosive power of lower body muscles	CMR	PLA	236.833*	0.000	116.781–356.886
		CG	70.278	0.351	-42.679–183.234
	CG	PLA	166.556*	0.001	69.422–263.689
Explosive power of upper body muscles	CMR	PLA	9.056	0.198	-3.184–21.295
		CG	-0.611	1.000	-16.533–15.310
	CG	PLA	9.667	0.491	-7.965–27.299
Speed of movement	CMR	PLA	-0.614*	0.000	-0.899 – -0.329
		CG	-0.201*	0.012	-0.361 – -0.040
	CG	PLA	-0.413*	0.001	-0.656 – -0.171
Isometric hand strength	CMR	PLA	1.576	0.070	1.539–9.905
		CG	1.399	1.000	-0.213–7.213
	CG	PLA	1.292	0.089	-1.209–5.654
Serv	CMR	PLA	2.556*	0.005	0.744–4.367
		CG	0.778	0.939	-1.208–2.763
	CG	PLA	1.778	0.211	-0.665–4.221
Forehand drive	CMR	PLA	1.722*	0.003	0.570–2.874
		CG	1.333	0.071	-0.090–2.757
	CG	PLA	0.389	1.000	-0.919–1.697
Backhand Push	CMR	PLA	0.250	0.782	-0.320–0.820
		CG	0.500	0.554	-0.460–1.460
	CG	PLA	-0.250	1.000	-1.275–0.775
Counter	CMR	PLA	0.111	1.000	-0.629–0.851
		CG	0.278	1.000	-0.834–1.389
	CG	PLA	-0.167	1.000	-0.948–0.615
Cognitive test	CMR	PLA	2.500*	0.000	1.155 – -3.845
		CG	0.167	1.000	-0.699–1.032
	CG	PLA	2.333*	0.000	1.100–3.566

*The mean difference is significant at the .05 level. PLA: placebo. CMR: Caffeine mouth rinsing. CG: Caffeine chewing gum.

*: Significant difference compared to PLA(*P* < 0.05). #: Significant difference compared to CMR(*P* < 0.05).

PLA: placebo. CMR: Caffeine mouth rinsing. CG: Caffeine chewing gum.

## 5. Discussion

This research was conducted to investigate the cross-consumption of caffeinated gum and coffee mouth rinsing on the skill, cognitive and performance indicators of female table tennis players. We hypothesized that CMR and CG can affect the central nervous, respiratory, cardiovascular and skeletal-muscular systems due to the inhibition of adenosine receptors of the central nervous system and the same distribution in the intracellular fluid [7]. These characteristics of caffeine delay fatigue and accelerate the breakdown of fats and increase the level of free fatty acid in plasma, increase contractility and epinephrine secretion, reduce pain, and also improve reaction time [7]. For this reason, it seems that caffeine can improve the performance, skill and cognitive indicators of female table tennis players.

Seven indicators containing agility, movement speed, hand movement speed, hand-eye coordination, explosive power of lower and upper body muscles, and isometric handgrip strength were measured and analyzed to check the performance of the participants. Agility and also movement speed increased significantly in CMR and CG compared to placebo conditions. Also, movement speed had a significant increase in CG compared to the CMR condition. Some previous studies have confirmed the positive effect of caffeine on the improvement of agility and movement speed. For example, the results of a study showed that caffeine consumption improves speed, agility and heart rate in college students [33]. However, some studies have shown that caffeine consumption does not affect the agility and movement speed of athletes. A study showed that consuming 150 mg of caffeine (i.e. 2.3 mg/kg of body weight) with chewing gum had no performance advantage on the results of functional basketball tests such as speed and agility tests [34]. Differences in exercise protocols and caffeine supplementation strategies may contribute to the heterogeneity of the results.

In addition, we had a significant improvement in hand movement speed in CMR and CG conditions compared to placebo. No previous research was found to investigate the effect of caffeine on hand movement speed. However, the improvement of hand movement speed in this study can be explained by some previous evidence about agility [33]. Furthermore, the coordination of participants had increased in HWTT in CMR compared to the placebo condition. In this regard, there are consistent and inconsistent studies. For example, a study observed that consuming caffeine in moderate doses leads to strengthening memory and motor coordination performance [35]. But a study showed that rinsing the mouth with espresso coffee did not affect the attention and hand-eye coordination of active adults [36]. Differences in exercise fields and caffeine supplementation dosage may contribute to the heterogeneity of the results.

The effects of caffeine on performance are related to both central and peripheral mechanisms. It has been found that the effect of caffeine on the central nervous system (CNS) is related to the blocking of adenosine receptors, which prevents the decrease in nerve activity and the subsequent increase in muscle absorption [37]. Also, caffeine affects the CNS by stimulating the release of serotonin in the cerebral cortex, enhancing the function of the sympathetic system and reducing the activity of inhibitory neurons [38–40]. In addition, caffeine inhibits the activity of phosphodiesterase and as a result, increases plasma catecholamine and glycolysis activity and also increases the availability of energy for active muscles during exercise [41]. As a result of central and peripheral effects, it can be said that caffeine improves performance, which includes psychomotor performance, such as agility and decision-making accuracy [42,43]. It can probably be said that this increase in alertness and attention level due to stimulation of the central nervous system delays fatigue and has the potential to increase performance. This can lead to improvements in overall running speed, agility, and improvements in cognitive processing and coordination.

As another result, the explosive power of the lower body muscles of participants increased significantly in CMR and CG conditions compared to placebo. The positive effect of caffeine on explosive power had been observed in some previous studies. For example, a relatively low absolute dose of caffeine (300 mg) given as gum, 10 min before exercise, has been reported to be effective for acute improvements in vertical jump height, lower body muscle strength, and total body strength in resistance-trained men [44]. Also, repeated CMR improved vertical jump performance in futsal players [15]. However, some studies showed inconsistent results. The combination of a low dose of caffeine with carbohydrates did not improve the relative peak power and increase the height of the vertical jump [45]. Probably, the difference in the type and dosage of supplements as well as the gender of the participants can be the reasons for these contradictory findings with the present study.

The explosive power of upper body muscles and isometric handgrip strength improved by CMR and CG compared to the placebo condition but these improvements were not significant. Consistently with these results, a study showed that consuming 150 mg of caffeine as chewing gum had no significant effect on the power and isometric handgrip strength of basketball players [34]. However, inconsistency with the present results, it had been shown in a meta-analysis that caffeine consumption increased the throwing performance of athletes [29]. The proposed mechanisms that may explain the energizing effects of caffeine are improving neuromuscular function, increasing endorphin secretion, improving alertness and reducing activity perception during exercise [46]. Caffeine energization has effects on the muscle that can directly help it. Probably, the increase in the formation of cross-muscular bridges leads to the production of more force, and hence more strength and power can be produced. The most probable way that caffeine can be useful for muscle contraction is through the mobility of calcium ions (Ca^2+^), which facilitates the production of force by each motor unit [47,48]. Therefore, it can be said that the speed of calcium release from the sarcoplasmic reticulum following caffeine consumption also increases muscle contraction force [49] in the same way; Caffeine partially increases the activity of the sodium/potassium (Na/K) pump and potentially increases the stimulation and contraction required for muscle contraction. Probably, this mechanism can be effective in strength performance and muscle force production, reducing reaction time and increasing speed [50]. So, it seems that the improvement of the explosive power of lower and upper body muscles and isometric handgrip strength can be due to these reasons. It can be predicted that if the number of participants was more, the increase of the explosive power of upper body muscles and isometric handgrip strength could be significant.

Four skill tests containing service, backhand push, counter and forehand drive were measured to check the skill of the participants. The results showed that serving and forehand drive skills had a significant improvement in CMR condition compared to placebo. Some previous studies confirmed these results. For example, In a research, it was observed that the consumption of 6 mg of caffeine improves successful shots and increases the accuracy of tennis player’s serve [51,52]. However, some studies showed other opposite results. Two studies showed that caffeine consumption does not have a significant effect on the speed and accuracy of the serve in tennis [53,54]. Also, 2 and 4 mg of caffeine per body weight produced no differences in shooting accuracy, response time, or target tracking time between groups [55].

About other findings, we had no significant increase in the backhand push and counter tests between conditions. There are some studies with the same results compared to the present study. Consuming 150 mg of caffeine had no performance advantage on the results of skill-based tests in elite female basketball players [34]. However, a study revealed that caffeine consumption via caffeinated gum significantly improved attack accuracy compared to non-caffeinated gum (placebo) [56]. It seems that the small number of participants compared to the mentioned research is the reason for this difference.

Also, the result of the cognitive test showed a significant improvement in CMR and CG compared to placebo conditions. In this regard, receiving 6 mg of caffeine 30 minutes before the training protocol in male amateur paddle tennis players increased the percentage of correct strokes and reduced errors [21]. It seems that the difference in supplementation strategy can be the reason for this inconsistent result. Also, no consistent result was found with the present result.

So, as a result, it seems that the improvement and increase of performance, skill and cognitive indicators of table tennis players in the mentioned research as well as the present research, is because the time to reach the physiological peak with the administration of caffeinated gum is very fast. Because caffeine enters the bloodstream through the capillaries of the mouth and therefore bypasses the digestive system. This rapid rate of absorption is a distinct advantage for athletes because the time between administration and absorption is very short. It is also possible that mouth rinsing with caffeine (duration 5–20 seconds) increases athletic performance due to activation of the sensory-motor cortex [57]. The mechanisms involved in the potential ergogenic effect of caffeine mouth rinsing are not fully understood, but two mechanisms have been proposed. The first mechanism involves the binding of caffeine with adenosine receptors located in the oral cavity, which increases the release of neurotransmitters and increases muscle contraction [16]. The second mechanism refers to bitter taste receptors in the mouth that are directly connected to areas of the brain associated with information processing and reward [58,59]. These receptors, in turn, are activated upon exposure to caffeine and improve mental alertness through dopamine transmission by activating sensory neurons in the mouth, initiating a cascade of transmission events to the brain [11].

It is important to acknowledge the limitations of our investigation. Firstly, due to financial constraints, the authors of this study could not measure the changes in plasma levels of caffeine after coffee mouth rinsing and chewing caffeine gum. Additionally, since our participants were exclusively female table tennis players, caution should be taken when generalizing our findings to other athletes. Furthermore, it is essential to note that the sample size in this research was limited due to the size of a table tennis team, which usually has 4 to 6 players. Therefore, future studies should include the evaluation of these variables to expand on our findings.

## 6. Conclusion

In conclusion, according to the results of the present study, CMR and CG can be almost effective on table tennis players’ performance, skill and cognitive indicators. However, CMR and CG allegedly have a better effect on functional and cognitive tests than skill tests. In addition, CMR appears to have more profound effects on performance improvement than CG. Additionally, according to the findings of the present study, it may be beneficial for table tennis players to incorporate CMR and CG techniques prior to the start of a match and during timeouts between rallies and games to enhance their performance. Also, future studies should focus on identifying the underlying mechanisms of CMR and CG effects on sports performance.
